# Editorial: Integration of physical activity for academic excellence and health promotion in adolescents and school children

**DOI:** 10.3389/fspor.2026.1986654

**Published:** 2026-10-23

**Authors:** Souhail Hermassi, Sascha Ketelhut, Roland van den Tillaar, Abdulla Alneama

**Affiliations:** 1 Department of Physical Education, College of Education, Qatar University, Doha, Qatar; 2 Institute of Sport Science, University of Bern, Bern, Switzerland; 3 Department of Sports Science and Physical Education, Nord University, Levanger, Norway; 4 Al Arabi Sports Club, Doha, Qatar

**Keywords:** academic achievement, children and adolescents, motor competence, physical activity, physical literacy, school-based health promotion

## 1 Introduction

Regular physical activity is widely recognized as a cornerstone of healthy growth and development in children and adolescents. Beyond its physical health benefits, evidence indicates that physical activity contributes to cognitive development, academic achievement, mental health, social competence, and lifelong healthy behaviors (1–3). At the same time, increasing physical inactivity, sedentary behavior, childhood obesity, and declining physical fitness represent major global public health concerns (4, 5).

Schools provide a unique setting in which education and health promotion intersect. Through high-quality physical education, physically active learning, and supportive school environments, they offer important opportunities to integrate movement into daily learning experiences. Consequently, there is growing interest in identifying strategies that simultaneously enhance academic achievement and support children's physical, psychological, and social well-being.

This Research Topic was launched to bring together multidisciplinary perspectives on how physical activity contributes to academic achievement, learning outcomes, and holistic child development. The thirteen contributions span physically active learning, teacher education, physical literacy, motor competence, health promotion, school policy, and child development. Collectively, they highlight the broader role of physical activity as a catalyst for cognitive development, educational success, and lifelong well-being.

## 2 Integration of the contributing articles

### 2.1 Physical activity, academic achievement, and learning

A major theme emerging from this Research Topic is the role of physical activity in supporting academic achievement and learning outcomes. The systematic review and meta-analysis by He et al. synthesized evidence demonstrating that school-based physical activity interventions can contribute positively to both academic achievement and learning outcomes. Complementing these findings, Che et al. highlighted the importance of self-regulation and self-efficacy as psychological mechanisms linking physical activity to academic achievement, while also identifying gender and urban-rural differences in these relationships.

The Research Topic also emphasizes that the educational benefits of physical activity extend beyond achievement measures. Physical activity can enhance engagement, self-regulation, and learning experiences, supporting a more holistic understanding of educational success. This perspective is particularly evident in studies focusing on physically active learning and teacher education. Lander et al. examined the integration of physically active learning within initial teacher education. In a separate study, Lander et al. introduced an observational instrument for evaluating physically active teaching strategies. Similarly, Daly-Smith et al. highlighted the capabilities, agency, and professional support required for teachers to successfully implement and sustain physically active learning in schools. Together, these studies underscore the importance of both individual teacher competencies and system-level support for embedding movement into educational practice.

A broader conceptual perspective is provided by Mandelid, who advocates a shift from physically active learning toward movement-centred pedagogy. This contribution challenges educators and researchers to reframe movement as an integral feature of children's learning experiences rather than simply a method for achieving educational outcomes.

### 2.2 Physical literacy, motor competence, and child development

The development of physical literacy and motor competence represents another important theme within the Research Topic. Tao et al. developed a structured framework for assessing physical literacy among preschool children, providing an important foundation for future evaluation and policy development. Chen et al. further contributed to this area by identifying profiles of motor coordination and health-related fitness among preschool children, highlighting developmental variability and the need for targeted interventions.

The relationship between movement and creativity was examined by Khorkova et al., whose findings suggest that innovative pedagogical approaches can simultaneously enhance cognitive creativity, motor creativity, and motor fitness. Wagner et al. extended this perspective by demonstrating relationships between muscular strength, neuromuscular control, and resilience, emphasizing the interconnected nature of physical and psychological development. Collectively, these studies reinforce the multidimensional role of physical activity in supporting children's cognitive, motor, and psychosocial growth.

### 2.3 Health promotion and school environments

Several contributions demonstrate the critical role of physical activity in promoting health and well-being within school settings. Li and Ma examined the effects of physical activity on body mass index, motor skills, and sleep patterns, illustrating the broad influence of active lifestyles on children's health and development.

Teachers also emerged as important agents of change. Kayitesi et al. highlighted the role educators can play in fostering healthy behaviors and supporting childhood obesity prevention initiatives. Beyond individual behaviors, school policies and environments were identified as influential factors shaping physical activity opportunities. Gardner et al. showed how a daily sport uniform policy can facilitate physical activity participation, enhance student comfort and well-being, and promote more inclusive and equitable school environments.

Taken together, these studies demonstrate that effective school-based health promotion requires coordinated action across multiple levels, encompassing children's behaviors, teacher practices, educational environments, and institutional policies. The findings support the development of comprehensive approaches that integrate physical activity into both educational and health-promotion frameworks.

Across all themes, a common message emerges: physical activity should be regarded as a fundamental component of children's educational experiences rather than an isolated health behavior. The collective evidence suggests that movement influences learning and development through interconnected physical, cognitive, psychological, social, and environmental pathways. By integrating findings from education, public health, sport science, and developmental research, this collection provides a strong scientific foundation for future school-based interventions and policies.

## 3 Future research directions

The articles included in this Research Topic highlight several priorities for future research. First, additional longitudinal and intervention studies are needed to strengthen the evidence regarding causal pathways linking physical activity, academic achievement, cognitive development, and health outcomes. Second, further work is required to evaluate the long-term implementation and sustainability of physically active learning across different educational systems. Third, continued refinement of physical literacy frameworks and assessment tools will be essential for evidence-based practice and policy development. Finally, future interdisciplinary research should examine how schools can more effectively integrate physical activity into everyday learning experiences while addressing cultural, socioeconomic, and contextual variations that influence implementation and outcomes.

## 4 Conclusion

Taken together, the thirteen contributions in this Research Topic provide compelling evidence that integrating physical activity into educational settings can support cognitive development, academic achievement, health promotion, and lifelong well-being. Across diverse methodologies and educational contexts, the studies highlight the value of movement as a key component of children's learning and development. Nevertheless, important challenges remain, including heterogeneity in definitions and assessment methods, differences across age groups and educational systems, limited longitudinal and experimental evidence, and uncertainties regarding causal mechanisms. Future research should also address the sustainability and scalability of school-based interventions and expand evidence from underrepresented cultural and geographical contexts. Despite these challenges, the collective findings support the view that physical activity should be considered a fundamental component of children's educational experiences. We hope this collection will stimulate future interdisciplinary research and inform evidence-based policies and practices that place movement at the heart of children's education.
